# Is primary trabeculectomy cost-effective for patients with advanced primary open angle glaucoma? Results from the Treatment of Advanced Glaucoma Study economic model

**DOI:** 10.1136/bjo-2023-323390

**Published:** 2024-02-09

**Authors:** Hosein Shabaninejad, Tara Homer, Ashleigh Kernohan, Anthony J King, Jennifer Burr, Augusto Azuara-Blanco, Luke Vale

**Affiliations:** 1 Newcastle University Population Health Sciences Institute, Newcastle upon Tyne, UK; 2 Ophthalmology, Nottingham University Hospitals NHS Trust, Nottingham, UK; 3 University of St Andrews School of Medicine, St Andrews, UK; 4 Centre for Public Health, Queen's University Belfast Centre for Public Health, Belfast, UK

**Keywords:** Glaucoma, Treatment Surgery

## Abstract

**Background/aims:**

Advanced primary open angle glaucoma (POAG) is a lifelong condition. The aim of this study is to compare medical treatment against trabeculectomy for patients presenting with advanced POAG using an economic evaluation decision model.

**Methods:**

A Markov model was used to compare the two treatments, medical treatment versus trabeculectomy for the management of advanced POAG, in terms of costs and quality-adjusted life-years (QALYs). The uncertainty surrounding the model findings was assessed using probabilistic sensitivity analysis and deterministic analysis. Data for the model came from Treatment of Advanced Glaucoma Study supplemented with data from the literature. The main outcomes of the model presented in terms of Incremental costs and QALYs based on responses to the EQ-5D-5L, Health Utilities Index-3 and a Glaucoma Utility Index.

**Results:**

In the base-case analysis (lifetime horizon and EQ-5D-5L measure), participants receiving trabeculectomy had on average, an additional cost of £2687, an additional 0.28 QALYs and an incremental cost per QALY of £9679 compared with medical treatment. There was a 73% likelihood of trabeculectomy being considered cost-effective when society was willing to pay £20 000 for a QALY. Over shorter time horizons, the incremental cost per QALY gained from trabeculectomy compared with medical treatment was higher (47 663) for a 2-year time horizon. Our results are robust to changes in the key assumptions and input parameters values.

**Conclusion:**

In patients presenting with advanced POAG, trabeculectomy has a higher probability of being cost-effective over a patient’s lifetime compared with medical treatment.

**WHAT IS ALREADY KNOWN ON THIS TOPIC**

- The impact of advance glaucoma on patients is lifelong.
- Existing economic evaluations studies have been in patients with early glaucoma, but there is a lack of evidence of the longer-term impact of surgery versus medication for patients with advanced glaucoma.

**WHAT THIS STUDY ADDS**

- Surgery is likely to be considered a cost-effective strategy compared with medical treatment in patients presenting with advanced glaucoma.

**HOW THIS STUDY MIGHT AFFECT RESEARCH, PRACTICE OR POLICY**

- This study provides the lifetime impact of interventions in patients with advanced glaucoma using an economic model. Longer-term follow-up trial studies (ie, 10 years) are required to collate the impact of interventions in the real world.

## Introduction

Primary open angle glaucoma (POAG) is a chronic irreversible optic neuropathy.^1^ The risk of POAG is higher among older people with 2% and 10% of people over the age of 40 and 80, respectively, in the UK having the disease.^2^ Given the ageing population, the number of people with POAG will increase^3^ and they are likely to live longer with their condition.

Glaucoma is the main reason for irreversible blindness globally.^1^ It is usually asymptomatic in its early stages. In the UK, 10%–39% of glaucoma is advanced in at least one eye at presentation (diagnosis).^4^ More advanced glaucoma increases morbidity and mortality, increases the risk of blindness and reduces quality of life.^5^ The severity of glaucoma increases healthcare-related costs for disease treatment and increases the risk of other health problems such as fractures.^6^ While effective treatment cannot cure glaucoma, it can slow or arrest progression and prevent vision loss and potential blindness.^7^

Reducing intraocular pressure (IOP) is the only effective treatment to prevent progression of glaucoma.^8^ In the UK, the National Institute for Health and Care Excellence recommend primary augmented trabeculectomy surgery, but acknowledged the lack of evidence (particularly economic) supporting this recommendation, the European Glaucoma Society also recommends primary trabeculectomy.^9^ A Cochrane review concluded that trabeculectomy produces better IOP reduction than medical treatments, but indicated uncertainty as to which therapy, was more effective and cost-effective for patients presenting with advanced glaucoma.^8^ The last trial which compared primary medical and surgical management of glaucoma (which excluded people with severe disease) recruited patients over 25 years ago^10^ and does not reflect either modern medical or surgical treatment. Since then, further glaucoma medications have become available which are believed to be more effective and less costly.^11^ Trabeculectomy has evolved and arguably provides better safety and efficacy outcomes than previously.^12^ From the perspective of the health service, medical treatment avoids the initial cost of surgery but may be associated with more visual field loss in the longer term. In part, this may be caused by non-adherence, as adherence to medication in the long term may be challenging for patients.^13^ Furthermore, there are the continuing costs of lifelong medication. It is also possible that some patients who begin pharmacological treatment may also require future trabeculectomy if medical treatment does not result in accurate control of IOP and vice versa.

## Materials and methods

The Treatment of Advanced Glaucoma Study (TAGS) was undertaken to address uncertainty around the effectiveness, safety and cost-effectiveness of primary trabeculectomy compared with medical treatment as treatments for those with advanced POAG. The TAGS study is a randomised controlled trial that compares two treatment approaches in terms of clinical outcomes, patient-reported outcomes and health service outcomes. While the primary intervention follows the study protocol, all other care is determined by the participant’s clinician, aligning with standard clinical decision-making within the typical clinical setting. The results of TAGS showed trabeculectomy lowered IOP but at 2 years there was no evidence of a difference in health-related quality of life.^14^ This latter finding is not surprising at 2 years given the generally slow progression of treated glaucoma. However, it is possible that in the longer term the differences in the control of IOP and further management requirements may result in differences in health-related quality of life. For example, medications are required in the long term and a longer follow-up allows more time for the costs of medication to offset the costs of surgery. In this paper, we consider what the costs, effects and cost-effectiveness outcomes are in the long term. To do this, we report an economic evaluation Markov model to extrapolate the results of the trial beyond the 2-year follow-up and up to the expected lifetime of patients.

### Model structure

**Figure 1:**
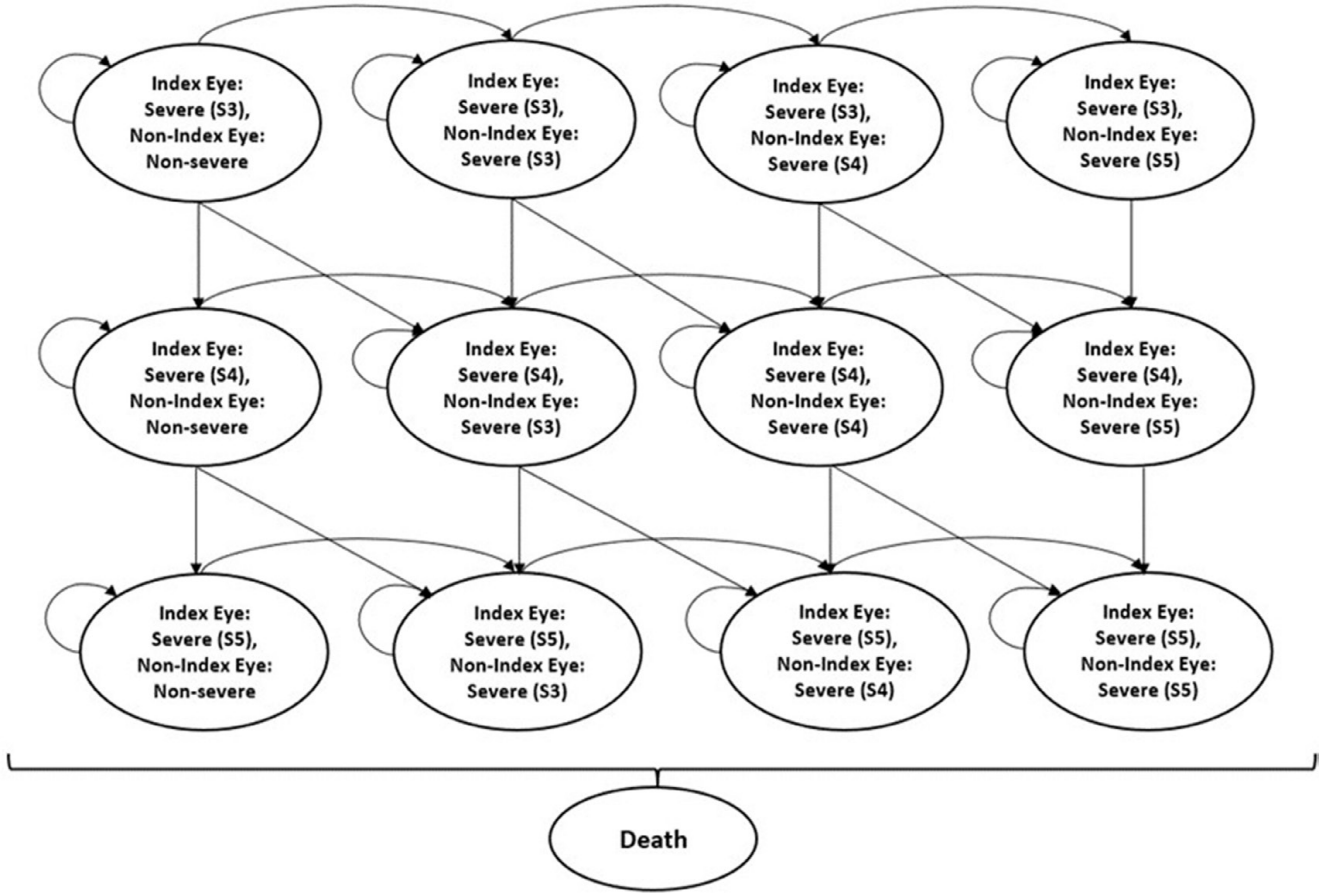
Markov model structure.

A Markov model was developed using experience drawn from previous evaluations of glaucoma treatment.^15–18^ Markov models capture the costs and benefits of treating chronic medical conditions such as glaucoma over time.^19^ The model simulates the patient pathway from initial treatment until death. The patient pathway described by the Markov model involves a series of mutually exclusive states that a patient can move between over time (figure 1). Once someone is in a state then they stay in that state for a define period of time called the cycle length. At the end of the cycle length, a patient moves to another state, or potentially stay in the same state if that is clinically plausible. Movement (transitions) between states is governed by a set of transition probabilities. Each Markov model includes at least one absorbing state. This is a state that a person can enter but cannot leave. In the context of a chronic disease, the absorbing state might be death.^20^ Mirroring the TAGS participant’s characteristics, the model starts with patients having advanced disease in one or both eyes and needing treatment. Over time, the glaucoma may progress, with some patients moving to more severe levels of glaucoma. Again, mirroring the trial inclusion criteria, the eye with advanced glaucoma according to Hodapp criteria was defined as the index eye in terms of initial treatment. In participants with both eyes eligible, the index eye was defined as the eye with the least severe disease defined by the mean deviation of the Humphrey visual field test in dB. We have used the (modified) glaucoma disease severity classification (GSS2)^21^ to define the stages of disease severity for the economic model (figure 1). Within the model, the Markov states represent, from left to right, increasing disease severity in each eye. All the programming for the model was implemented in TreeAge Pro.^22^

We have adopted this model structure to model both eyes independently and estimate the chance of unilateral and bilateral blindness more precisely. This is especially important in the case of eye disease as health-related quality of life and hence quality-adjusted life-years (QALYs) are thought to be determined by the quality of vision in the better eye.^23^ The model structure was developed in collaboration with clinical experts from the TAGS research team to ensure validity. To establish external consistency, the model results were compared with outcomes reported in other trials and other economic evaluations.^15–17^

Participants can start in any of these states defined in figure 1 according to the severity state of their disease. In a particular model cycle, patients can remain within their existing state of health, or progress towards increased disease severity. Most Markov models would allow for multidirectional transitional movement, but in the case of an irreversible disease, such as glaucoma,^19^ a unidirectional movement (towards a worse disease severity) is the only transition possible. Markov’s tracings of disease states over time are provided in online supplemental appendix 1 figure S1.

10.1136/bjo-2023-323390.supp1 Supplementary data

The cycle length in our model is defined as 1 year. This is due to glaucoma’s relatively slow progression.^19^ The time horizon is the estimated lifetime of a patient in the 60s as this is the average starting age of participants in the TAGS trial. The transition probabilities between states were informed mainly by the data from TAGS trial as described in the next section. It is assumed that patients move sequentially between states and because of the relatively slow evolution of the glaucoma disease cannot skip states. Finally, death from all causes is included in the model as a single state. Transition probabilities to this state are assumed to be independent of severity and treatment history and are derived from age-specific/sex-specific life-tables.^24^ Patients who are blind (in one or both eyes) are taken to have a higher risk of death^25^ and hence standardised mortality ratios were used to adjust the risk of death for those who are blind in one or both eyes. More details on initial and transition probabilities are provided in online supplemental appendix 2 tables S1–S9.

### Model parameters

The economic evaluation analysis takes the perspective of the UK National Health Service and personal and social services. For extrapolation, the cost and utility values beyond the duration of the TAGs trial follow-up^26^ were assumed to vary according to clinical severity state and treatment allocation using linear regression models. Costs assigned to each state in the model, reflect the costs that would be incurred for each 12-month period. Outcomes are expressed in QALYs, which measure both quantity and quality of life. The mean QALYs for each intervention were calculated by multiplying amount of time patients spend in each health state by their utility values. Estimated utilities used in the model were based on TAGS data using the following preference-based instruments: EQ-5D-5L, Health Utilities Index (HUI-3) and the Glaucoma Utility Index (GUI). The EQ-5D-5L and HUI-3 are validated instruments, which provide a generic measure of health-related quality of life. They are short questionnaires from which utility values can be derived from existing tariffs.^27 28^ The EQ-5D-5L and the HUI-3 are not specifically designed to measure the quality of life of eye diseases although, the HUI-3 does have a specific question which asks about vision. The GUI is a condition-specific measure but used utilities derived directly from the TAGS population because relatively few of those contributing to the original GUI tariff had advanced glaucoma.^29^ All future costs and utilities used in the model were discounted at 3.5% per annum, the UK recommended rate, as the duration of follow-up (time horizon) was greater than 1 year.^30^ The results of the model are presented as average total costs, and average total QALYs for each utility measure for each of the interventions and incremental cost-effectiveness ratios (ICER).

### Sensitivity analysis

The main model inputs were varied in the sensitivity analyses to explore parameter and other forms of uncertainty surrounding model-based estimates to determine whether they may affect the ICER of trabeculectomy compared with medical treatment. The results of these sensitivity analyses are depicted in a tornado diagram to identify main inputs that could be altered to make trabeculectomy more or less cost-effective relative to medical treatment and presenting the results in a 2-year and 10-year time horizon.

All model input parameters (eg, cost and utility values) are defined as statistical distributions to facilitate probabilistic sensitivity analysis (PSA).^20^ The input parameters of the model were based on a data set where multiple imputations (MI) were performed for missing data. These data were used to parameterise uncertainty surrounding the joint incremental costs and effects which are presented graphically as confidence ellipses on the incremental cost-effectiveness plane. Ranges and distributional assumptions for input parameters were based on the TAGS data^26^ assigned gamma distributions for costs and beta distributions for utility data.^20^ We also calculated correlations between the coefficients of cost and utility for the variables included in the time-to-event and logistic regression analyses using Cholesky decomposition and assigned multinormal distributions to these parameters in the model to account for uncertainty in the estimated transition probabilities. The results of PSA are presented as a cost-effectiveness acceptability curve (CEAC). The CEAC summarises the impact of uncertainty using Monte Carlo simulation where the model is analysed 10 000 times choosing random values from its assigned distributions for each input parameter.

## Results

### Incremental costs, QALYs and cost-effectiveness

Table 1 shows the QALYs (for EQ-5D-5L, HUI-3 and GUI presented separately), total cost and incremental cost per QALY. In the base-case analysis (lifetime horizon and EQ-5D-5L-based QALYs), trabeculectomy had, on average, an additional cost of £2687, an additional 0.28 QALYs, and an incremental cost per QALY gained of £9679 compared with medical treatment. The results of the PSA showed, should society be willing to pay £20 000 per QALY,^31^ that the likelihood of trabeculectomy being cost-effective compared with medical treatment is 73%.

**Table 1:** Incremental cost-effectiveness measures (model-based analysis)

Time horizon	Intervention	Cost (£)	Δ Cost (£)	QALY	ΔQALY	ICER (Δ Cost/ΔQALY) (£)	NMB	Probability cost-effective at Rc			
								0	£10 000	£20 000	£50 000
EQ-5D-based QALYs											
2-year time horizon	Trabeculectomy	3436	2106	1.23	0.04	47 663	21 108	0.1	0.26	0.39	0.5
	Medication	1330		1.18			22 330	0.9	0.74	0.61	0.5
10-year time horizon	Trabeculectomy	5421	2362	3.72	0.17	13 911	69 024	0.08	0.4	0.59	0.73
	Medication	3059		3.55			67 990	0.92	0.6	0.41	0.27
Lifetime horizon	Trabeculectomy	7273	2687	5.92	0.28	9679	111 052	0.08	0.5	0.73	0.85
	Medication	4586		5.64			108 187	0.92	0.5	0.27	0.15
HUI-based QALYs											
2-year time horizon	Trabeculectomy	3436	2106	1.17	0.05	39 724	19 978	0.09	0.29	0.4	0.52
	Medication	1330		1.12			21 024	0.91	0.71	0.6	0.48
10-year time horizon	Trabeculectomy	5421	2362	3.58	0.22	10 506	66 115	0.1	0.5	0.65	0.75
	Medication	3059		3.35			63 980	0.9	0.5	0.35	0.25
Lifetime horizon	Trabeculectomy	7273	2687	5.70	0.38	7016	106 779	0.06	0.62	0.81	0.88
	Medication	4586		5.32			101 806	0.94	0.38	0.19	0.12
GUI-based QALYs											
2-year time horizon	Trabeculectomy	3436	2106	1.24	0.01	147 247	21 302	0.09	0.18	0.28	0.41
	Medication	1330		1.23			23 122	0.91	0.82	0.72	0.59
10-year time horizon	Trabeculectomy	5421	2362	3.73	0.1	24 179	69 269	0.08	0.26	0.44	0.6
	Medication	3059		3.64			69 677	0.92	0.74	0.56	0.4
Lifetime horizon	Trabeculectomy	7273	2687	5.92	0.16	16 805	111 165	0.06	0.32	0.55	0.74
	Medication	4586		5.76			110 655	0.94	0.68	0.45	0.26

EQ-5D, EuroQol Five Dimension; GUI, glaucoma utility index; HUI, health utilities index; ICER, incremental cost-effectiveness ratio; NMB, net monetary benefit; QALY, quality-adjusted life-year; Rc, ceiling ratio of willingness to pay per QALY gained.

The model-based estimates of mean costs and QALYs at 2 years indicate that trabeculectomy is expected to, on average, cost an additional £2106 for a QALY gain of 0.04 compared with medical treatment. The corresponding incremental cost per QALY for trabeculectomy compared with medical treatment would be of £47 663 (table 1). For a 10-year time-horizon again trabeculectomy is on average more costly (£2362) and more effective (0.17 QALYs) with an incremental cost per QALY gained of £13 911 and a 59% likelihood of being cost-effective compared with medical treatment at a threshold value of £20 000 per QALY.

Table 1 also reports the incremental cost, incremental QALY, incremental cost per QALY when QALYs are based on HUI-3 and GUI utility values. For lifetime horizon and HUI-3-based QALYs, trabeculectomy had, on average, an additional 0.38 QALYs, and an incremental cost per QALY gained of 7016 compared with medical treatment. The results of the PSA showed that the likelihood of trabeculectomy being cost-effective compared with medical treatment was 81% at £20 000 per QALY willingness to pay threshold. For GUI-based QALYs, trabeculectomy had, on average, an additional 0.16 QALYs, and an incremental cost per QALY gained of £16 805 compared with medical treatment. The results of the PSA showed that the likelihood of trabeculectomy being cost-effective compared with medical treatment was 55% at £20 000 per QALY willingness to pay threshold.

### Sensitivity analysis

**Figure 2:**
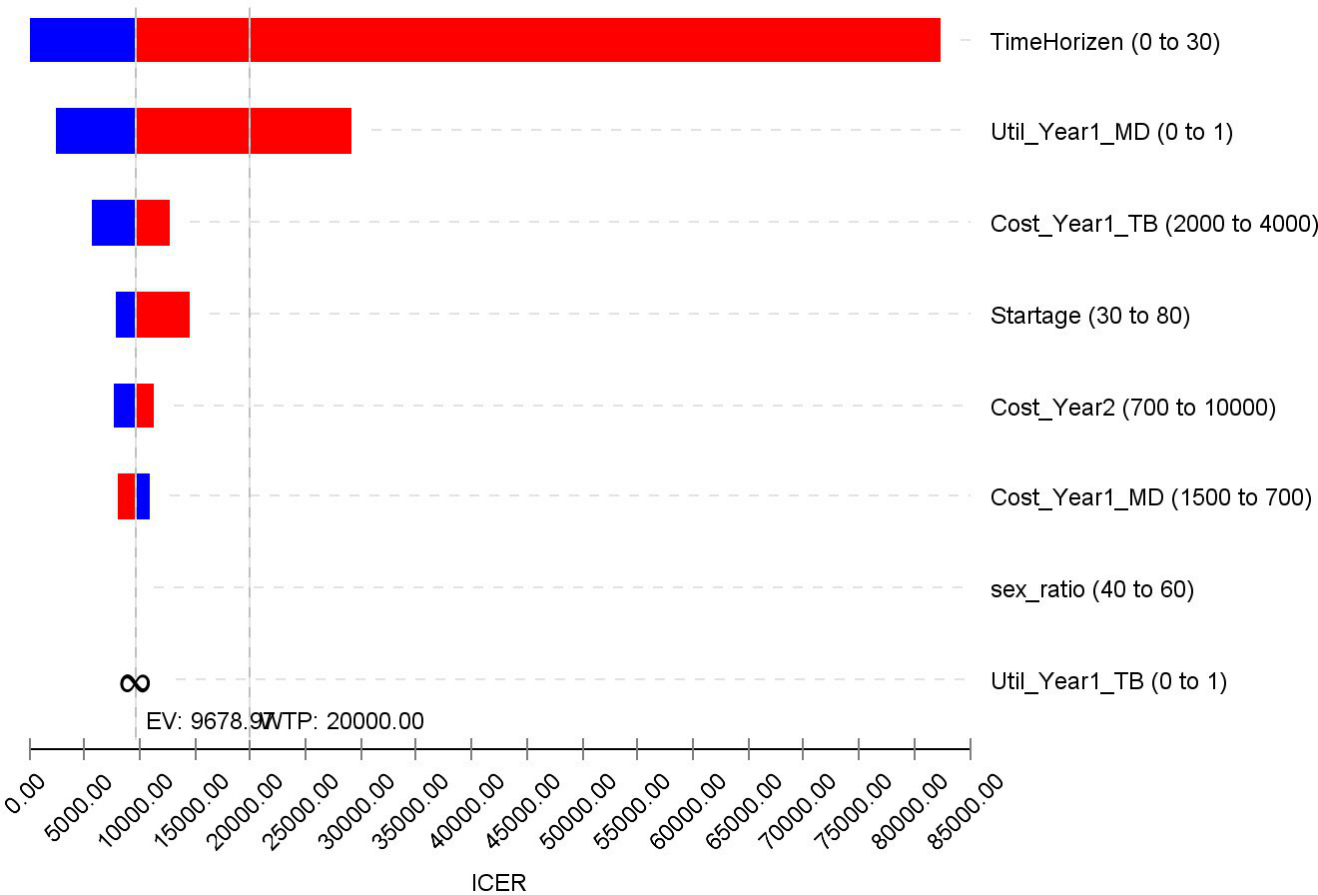
Tornado diagram for the main parameters (EQ-5D measure). EV, expected value; ICER, incremental cost-effectiveness ratio; WTP, willingness to pay; EQ-5D, EuroQol Five Dimension.

**Figure 3:**
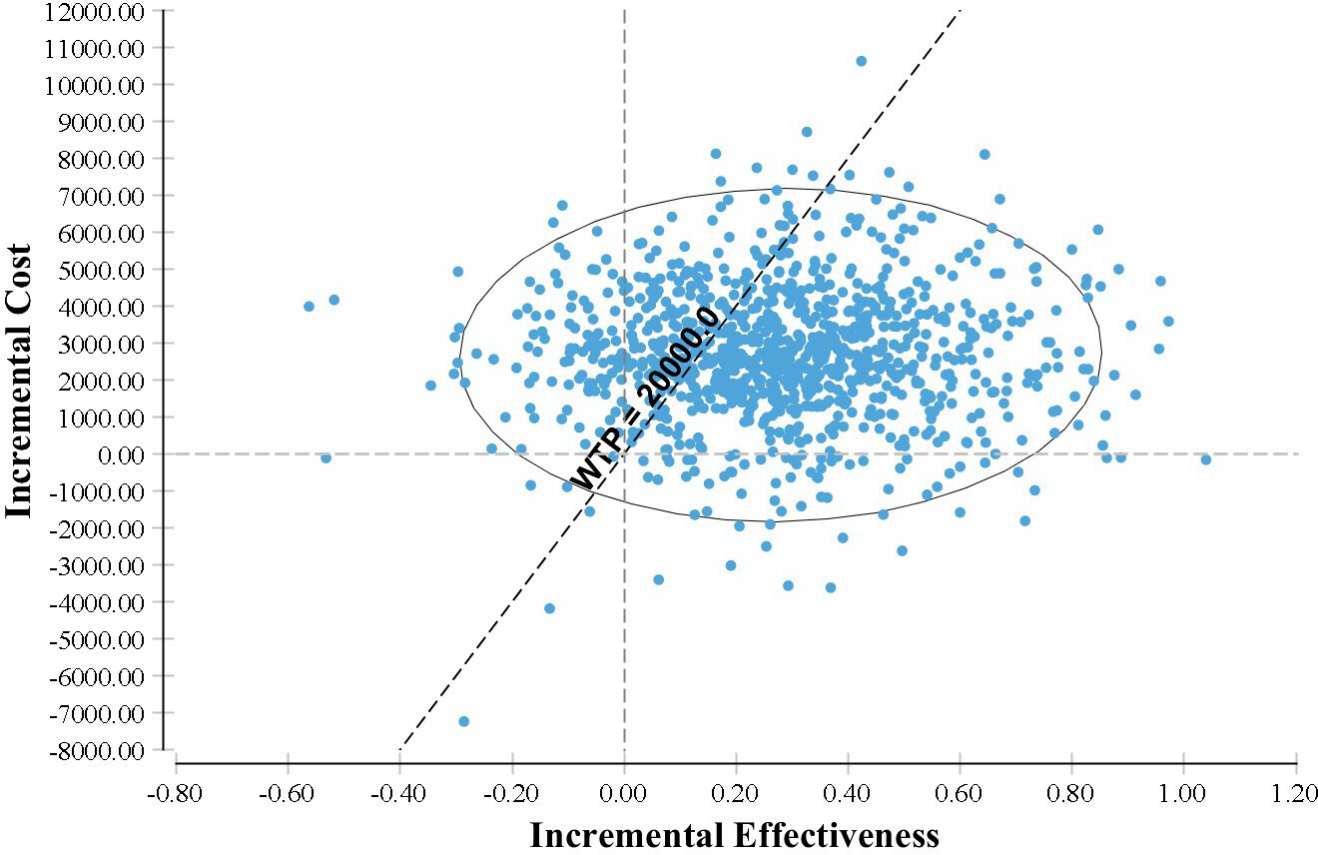
Incremental cost-effectiveness scatterplot; trabeculectomy versus medical treatment in the base-case analysis. WTP, willingness to pay.

**Figure 4:**
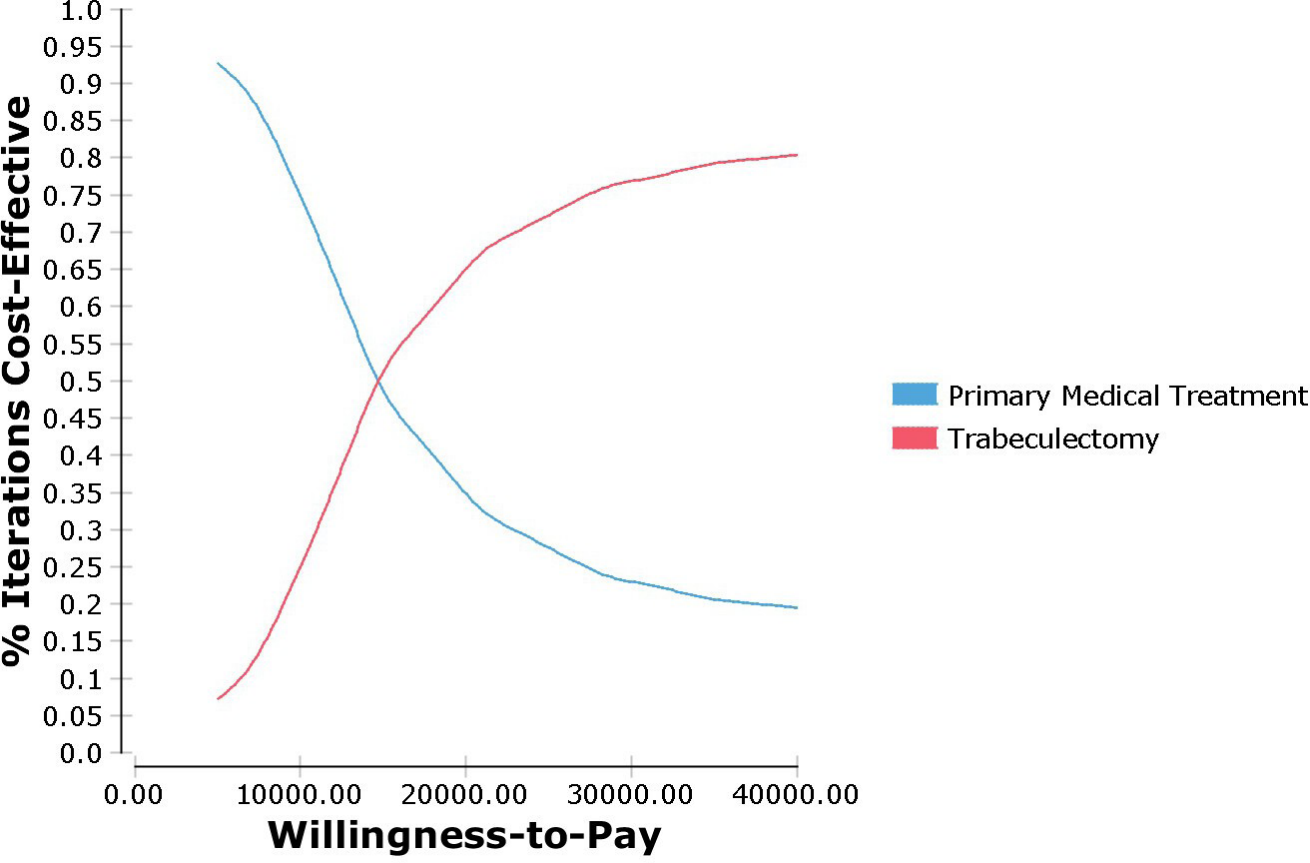
Model-based cost-effectiveness acceptability curve for the base-case analysis (lifetime horizon; EQ-5D measure). EQ-5D, EuroQol Five Dimension.

Deterministic sensitivity analysis shows that the model-based findings were generally robust to the changes examined (figure 2). As figure 2 shows length of the time defined in the model (time horizon) has the biggest effect if either of trabeculectomy or medical treatment to be cost-effective for EuroQol Five Dimension with 5 Levels (EQ-5D-5L) measure. Moreover, cost-effectiveness plane and the model-based CEAC based on the lifetime horizon presented in figure 3 and figure 4, respectively. More results of sensitivity analysis for other measures (HUI and GUI QALYs measures) are presented in online supplemental appendix 3 table S6 and figure S2–S7.

## Discussion

Our results suggest that on average trabeculectomy is more costly but more effective in terms of QALYs gained. Trabeculectomy is likely to be cost-effective compared with medical treatment in patients presenting with advanced glaucoma over the lifetime horizon over different values for our willingness to pay for an additional QALY. Although the initial costs (cost of year 1 treatment) are higher for patients assigned to trabeculectomy, these are partly offset in the longer term as these patients receive fewer subsequent procedures and have lower medication use.

While other economic evaluations exist, nearly all to date have been in a patient with early glaucoma,^16 17 32–34^ only one study has considered more severe glaucoma similar to TAGS. Guedes *et al*’s analysis used a Markov model to identify the most cost-effective treatment strategy for advanced glaucoma. The results of this study were similar to ours and they reported that medical treatment was not cost-effective compared with surgery for patients with advanced POAG over a lifetime horizon.^35^ In comparison to the study by Guedes *et al,* our model is more sophisticated as we considered each eye independently and our data came from a head-to-head trial-based comparison of trabeculectomy with medical treatment. Also, our data collected prospectively as part of a planned analyses in a pragmatic trial—rather than a hypothetical glaucoma population.

The TAGS-based analyses only considered patients with advanced glaucoma. Our evaluation has also considered the impact of using different methods to measure and value health-related quality of life. The advantage of this is that the EQ-5D-5L is widely used to estimate QALYs in many countries throughout the world. However, it does not include a specific vision component; something the HUI-3 does, while the GUI valuations are specific to advanced glaucoma.

When considering the different methods used to derive the QALYs; intuitively, it would be expected a generic health status instrument with a vision domain (HUI-3) to be more sensitive to visual outcomes than one which does not (EQ-5D-5L). The results of our study showed that using HUI-3 identified a larger QALYs difference between treatment methods (0.38 additional QALYs for trabeculectomy compared with medical treatment) compared with EQ-5D-5L (0.28 additional QALYs for trabeculectomy compared with medication). This is an important observation and suggests that when evaluating diseases affecting vision that it may be better to use the HUI-3 over EQ-5D-5L. The point should be considered that HUI-3 tariff is a based on an older sample that was based in Canada not the UK. So, the different population could drive the results as much as the inclusion/exclusion of a vision element. However, as there is a significant burden difference for patients and analysts in using the HUI-3 which is a much longer questionnaire than EQ-5D-5L this requires further evaluation.

Our study has some limitations. First was the limited data to extrapolate beyond 2 years for example, the need for further surgery (cataract or additional glaucoma surgery) in the longer term were not considered in the model as the TAGS within-trial results provided no evidence of a difference between two groups.^14^ For example, patients undergoing trabeculectomy may be more likely to require cataract surgery. Finally, to address uncertainty caused by missing data, we have used MI data for the model. The results of the MI suggest that the complete-case analysis may underestimate the difference in effects between the alternatives, as a result of those with poorer health outcomes being more likely to withdraw during the follow-up. Although there were no changes in conclusion while using complete case dataset and MI dataset.

In conclusion, our study suggests that surgery is likely to be considered a cost-effective strategy compared with medical treatment in patients presenting with advanced glaucoma in at least one eye over the patient’s lifetime.

## Data Availability

All data relevant to the study are included in the article or uploaded as online supplemental information.
