# Supplementary material for: Is primary trabeculectomy cost-effective for patients with advanced primary open angle glaucoma? Results from the Treatment of Advanced Glaucoma Study economic model
Source: Br J Ophthalmol. 2024 Feb 9;108(9):1210–5. doi: 10.1136/bjo-2023-323390 (PMC11347270; doi:10.1136/bjo-2023-323390)
Supplement: Supplementary data [file bjo-2023-323390supp001.pdf]

Supplement

Supplement

Appendix 1: Progression from one health states to another

We generated a year-by-year basis Markov tracing for both groups over a period of 30 years (Figure S1). This show the condition of patients at the start of the trial were mostly similar in both surgery and medication groups and most patients were at stage 2 (Index eye severe (S4) Non-index eye non-severe) of the disease at the start of the trial. Though based on, patients` treatment groups (medication or surgery) they have different trajectory during Markov model. For example, higher percentage of patients moved to stage 12 (both eye severe) and the percentage of patients at this stage after 12 years is about 25 percent, while in medication groups most patients moved to remained in stage 3 (one-eye severe).

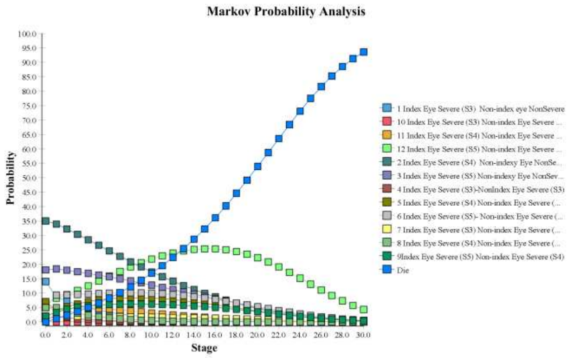

(a)Trabeculectomy

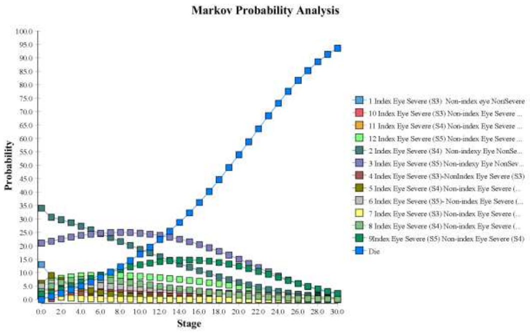

(b) Medication

Figure S1: Markov tracings of disease states over time for the (a) Trabeculectomy (b) Medication

Supplement

Appendix 2: Initial and transition probabilities of defined stages in the model

Table S1: Transition to the same and/or higher stages during 1st year in Surgery group (No (%))

	Baseline	stage1	stage2	stage3	stage4	stage5	stage6	stage7	stage8	stage9	stage 10	stage11	stage12
**stage1**	30	19 (0.63)	9 (0.3)		2 (0.07)								
**stage2**	76		65 (0.85)	8 (0.11)		1 (0.01)	2 (0.03)						
**stage3**	39			32 (0.82)			7 (0.18)						
**stage4**	2				2 (1)								
**stage5**	15					8 (0.54)	2 (0.13)		3 (0.2)	2 (0.13)			
**stage6**	11						10 (0.9)			1 (0.1)			
**stage7**	5							5 (1)					
**stage8**	10								7 (0.7)			1 (0.1)	2 (0.2)
**stage9**	4									4 (1)			
**stage10**	0												
**stage 11**	11											8 (0.73)	3 (0.27)
**stage12**	14												14 (1)
	**217**												

Shabaninejad H, *et al. Br J Ophthalmol* 2024; 108:1210–1215. doi: 10.1136/bjo-2023-323390

Supplement

Table S2: Transition to the same and/or higher stages during 1st year in Medication group (No (%))

	Baseline	stage1	stage2	stage3	stage4	stage5	stage6	stage7	stage8	stage9	stage 10	stage11	stage12
**stage1**	28	19 (0.68)	8 (0.29)			1 (0.03)							
**stage2**	74		59 (0.8)	3 (0.04)		9 (0.12)	3 (0.04)						
**stage3**	46			45 (0.98)			1 (0.02)						
**stage4**	3				1 (0.33)	1 (0.33)			1 (0.33)				
**stage5**	13					9 (0.7)	1 (0.07)		3 (0.23)				
**stage6**	10						8 (0.8)			2 (0.2)			
**stage7**	5							3 (0.6)	2 (0.4)				
**stage8**	6								5 (0.83)			1 (0.17)	
**stage9**	4									3 (0.75)			1 (0.25)
**stage10**	8										7 (0.88)	1 (0.12)	
**stage 11**	6											5 (0.83)	1 (0.17)
**stage12**	13												13 (1)
	**216**												

Shabaninejad H, *et al. Br J Ophthalmol* 2024; 108:1210–1215. doi: 10.1136/bjo-2023-323390

Supplement

Table S3: Transition to the same and/or higher stages during 2nd year in Surgery group (No (%))

	12Month	stage1	stage2	stage3	stage4	stage5	stage6	stage7	stage8	stage9	stage 10	stage11	stage12
stage1	19	16 (0.84)	3 (0.16)		0 (0)	0 (0)							
stage2	74		68 (0.92)	2 (0.03)		4 (0.05)	0						
stage3	40			37 (0.92)			3 (0.08)						
stage4	4				3 (0.75)	0 (0)		0 (0)	1 (0.25)				
stage5	10					9 (0.9)	1 (0.1)						
stage6	19						16 (0.84)			3 (0.16)			
stage7	5							5 (1)					
stage8	10								7 (0.7)	1 (0.1)		2 (0.2)	0 (0)
stage9	8									6 (0.75)			2 (0.25)
stage10	0										0 (0)		
stage 11	9											8 (0.89)	1 (0.11)
stage12	19												19 (1)
	**217**												

Shabaninejad H, *et al. Br J Ophthalmol* 2024; 108:1210–1215. doi: 10.1136/bjo-2023-323390

Supplement

Table S4: Transition to the same and/or higher stages during 2nd year in Medication group (No)

	12Month	stage1	stage2	stage3	stage4	stage5	stage6	stage7	stage8	stage9	stage 10	stage11	stage12
**stage1**	19	15 (0.8)	2 (0.1)		2 (0.1)								
**stage2**	66		63 (0.95)	3 (0.05)									
**stage3**	48			47 (0.98)			1 (0.02)						
**stage4**	1				1 (1)								
**stage5**	19					15 (0.79)	1 (0.05)		3 (0.16)				
**stage6**	13						11 (0.85)			2 (0.15)			
**stage7**	3							2 (0.67)	1 (0.33)				
**stage8**	12								11 (0.92)	1 (0.08)			
**stage9**	5									5 (1)			
**stage10**	7										7 (1)		
**stage 11**	7											5 (0.71)	2 (0.29)
**stage12**	15												15 (1)
	**215**												

Shabaninejad H, *et al. Br J Ophthalmol* 2024; 108:1210–1215. doi: 10.1136/bjo-2023-323390

Table S5: Initial & transition probabilities of stages in the model

**Trabeculectomy arm**					**Medication arm**				
		**Transition**					**Transition**		
**stages**	**initial**	**to**	**Year 1**	**Year 2**	**stages**	**initial**	**to**	**Year1**	**Year2**
**stage 1**	0.14	Stage 1 to 1	0.63	0.84	**stage 1**	0.13	Stage 1 to 1	0.68	0.79
		Stage 1 to 2	0.30	0.16			Stage 1 to 2	0.29	0.11
		Stage 1 to 4	0.07	0.00			Stage 1 to 4	0.00	0.11
		Stage 1 to 5	0.00	0.00			Stage 1 to 5	0.04	0.00
**stage 2**	0.35	Stage 2 to 2	0.86	0.92	**stage 2**	0.34	Stage 2 to 2	0.80	0.95
		Stage 2 to 3	0.11	0.03			Stage 2 to 3	0.04	0.05
		Stage 2 to 5	0.01	0.05			Stage 2 to 5	0.12	0.00
		Stage 2 to 6	0.03	0.00			Stage 2 to 6	0.04	0.00
**stage 3**	0.18	Stage 3 to 3	0.82	0.93	**stage 3**	0.21	Stage 3 to 3	0.98	0.98
		stage 3 to 6	0.18	0.08			stage 3 to 6	0.02	0.02
**stage 4**	0.01	stage 4 to 4	1.00	0.75	**stage 4**	0.01	stage 4 to 4	0.33	1.00
		stage 4 to 5	0.00	0.00			stage 4 to 5	0.33	0.00
		stage 4 to 7	0.00	0.00			stage 4 to 7	0.00	0.00
		stage 4 to 8	0.00	0.25			stage 4 to 8	0.33	0.00
**stage 5**	0.07	stage 5 to 5	0.53	0.90	**stage 5**	0.06	stage 5 to 5	0.69	0.79
		stage 5 to 6	0.13	0.10			stage 5 to 6	0.08	0.05
		stage 5 to 8	0.20	0.00			stage 5 to 8	0.23	0.16
		stage 5 to 9	0.13	0.00			stage 5 to 9	0.00	0.00
**stage 6**	0.05	stage 6 to 6	0.91	0.84	**stage 6**	0.05	stage 6 to 6	0.80	0.85
		stage 6 to 9	0.09	0.16			stage 6 to 9	0.20	0.15
**stage 7**	0.02	stage 7 to 7	1.00	1.00	**stage 7**	0.02	stage 7 to 7	0.60	0.67
		stage 7 to 8	0.00	0.00			stage 7 to 8	0.40	0.33
		stage 7 to 10	0.00	0.00			stage 7 to 10	0.00	0.00
		stage 7 to 11	0.00	0.00			stage 7 to 11	0.00	0.00
**stage 8**	0.05	stage 8 to 8	0.70	0.70	**stage 8**	0.03	stage 8 to 8	0.83	0.92
		stage 8 to 9	0.00	0.10			stage 8 to 9	0.00	0.08
		stage 8 to 11	0.10	0.20			stage 8 to 11	0.17	0.00
		stage 8 to 12	0.20	0.00			stage 8 to 12	0.00	0.00
**stage 9**	0.02	stage 9 to 9	1.00	0.75	**stage 9**	0.02	stage 9 to 9	0.75	1.00
		stage 9 to 12	0.00	0.25			stage 9 to 12	0.25	0.00
**stage 10**	0	stage 10 to 10	0.00	0.00	stage 10	0.04	stage 10 to 10	0.88	1.00
		stage 10 to 11	0.00	0.00			stage 10 to 11	0.12	0.00
**stage 11**	0.05	stage 11 to 11	0.73	0.89	stage 11	0.03	stage 11 to 11	0.83	0.71
		stage 11 to 12	0.27	0.11			stage 11 to 12	0.17	0.29
**stage 12**	0.06	stage 12 to 12	1	1	stage 12	0.06	stage 12 to 12	1	1

Appendix 3: SA results for GUI & HUI Measures

Table S6: Sensitivity Analyses of main parameters in the model

Parameters	value	ICER	ICER	ICER
Cost of year 1 (trabeculectomy)	2000	5594	4055	9713
	3000	9196	6666	15967
	4000	12798	9277	22222
Cost of year 1 (medication)	700	10986	7963	19076
	1100	9546	6919	16574
	1500	8105	5875	14072
Utility of year 1 (trabeculectomy)	0.2	Dominated	Dominated	Dominated
	0.6	70594	14483	Dominated
	1	6134	4589	8625
Utility of year 1 (medication)	0.2	3012	2819	3331
	0.6	5461	4856	6606
	1	29197	17528	395757
Cost of year 2	700	7726	5600	13415
	1100	7878	5711	13679
	1500	8031	5821	13944
Utility of year 2	0.2	52124	50146	96391
	0.5	34466	33590	49497
	1	20546	20231	25087
Time horizon, y<br>Age at (severe) glaucoma	2	47663	39724	147247
	10	13910	10506	24179
	30	9679	7016	16805
	30	7924	5614	13914
	42	8037	5703	14099

Shabaninejad H, et al. Br J Ophthalmol 2024; 108:1210–1215. doi: 10.1136/bjo-2023-323390

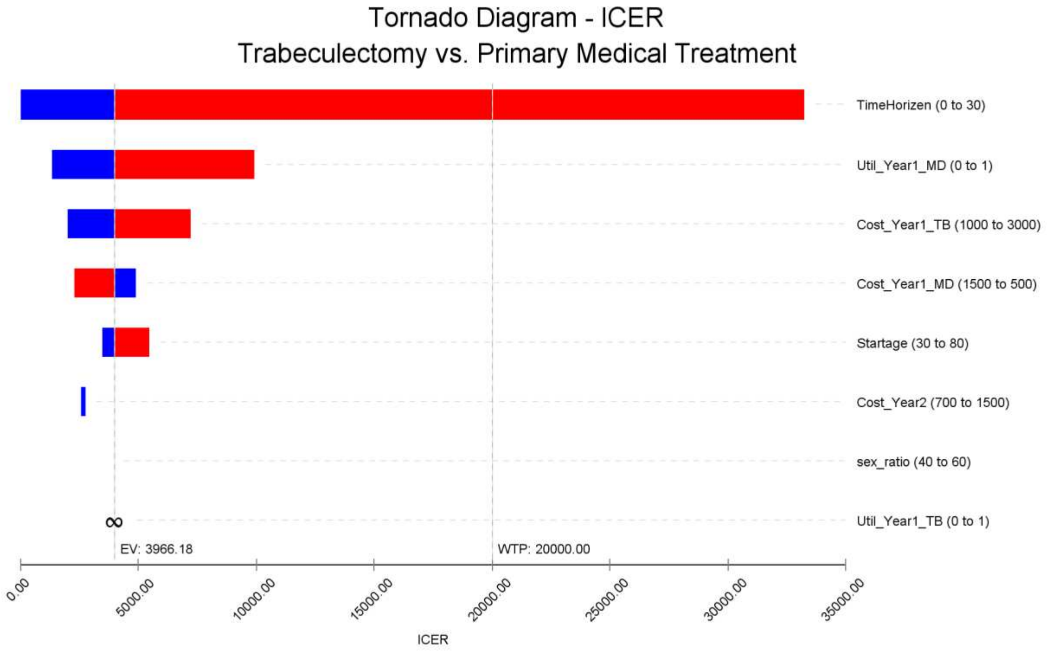

Figure S2: Tornado diagram for the main parameters (HUI measure) (EV: Expected value, WTP: Willingness to Pay)

Shabaninejad H, *et al. Br J Ophthalmol* 2024; 108:1210–1215. doi: 10.1136/bjo-2023-323390

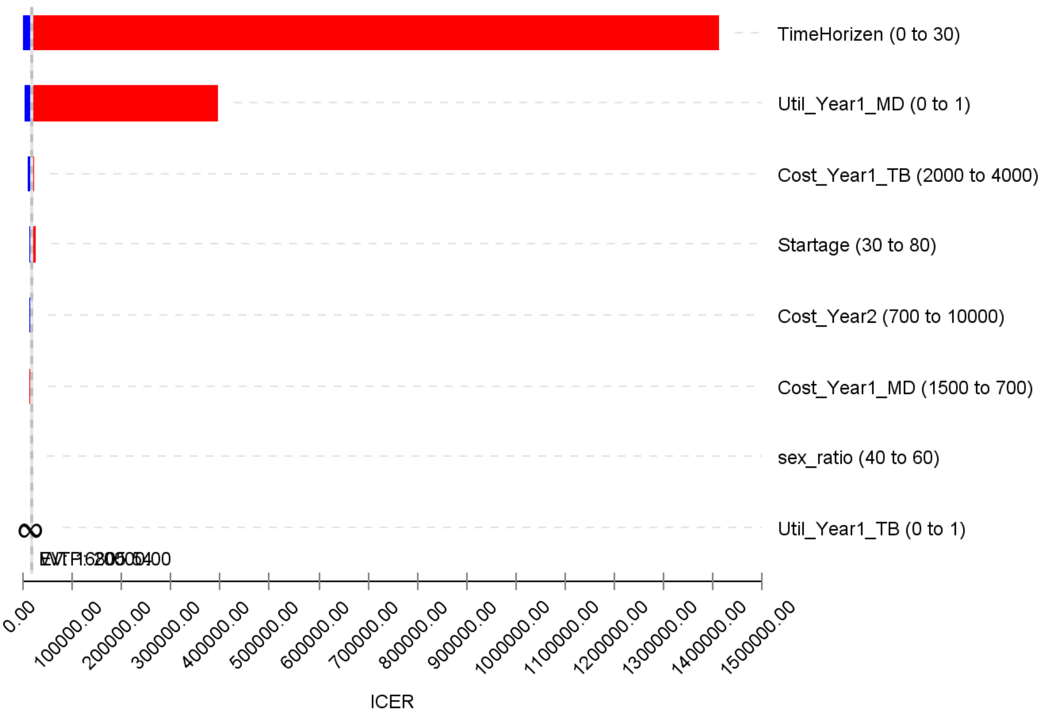

Figure S3: Tornado diagram for the main parameters (GUI measure) (EV: Expected value, WTP: Willingness to Pay)

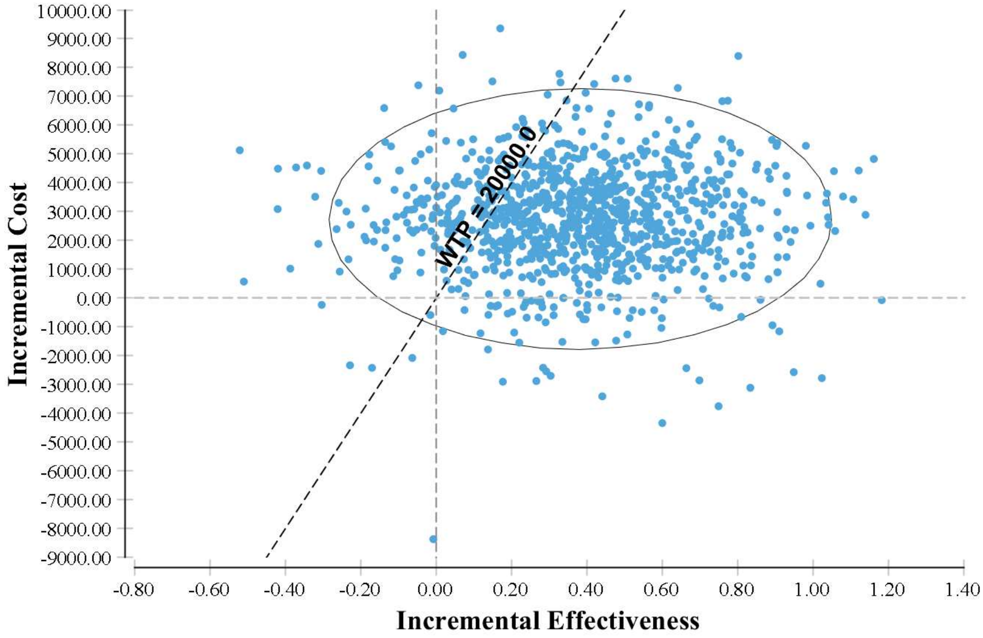

Figure S4: Incremental Cost-Effectiveness Scatterplot; Trabeculectomy vs Medical Treatment (HUI-3 measure) (WTP: Willingness to Pay)

Shabaninejad H, *et al. Br J Ophthalmol* 2024; 108:1210–1215. doi: 10.1136/bjo-2023-323390

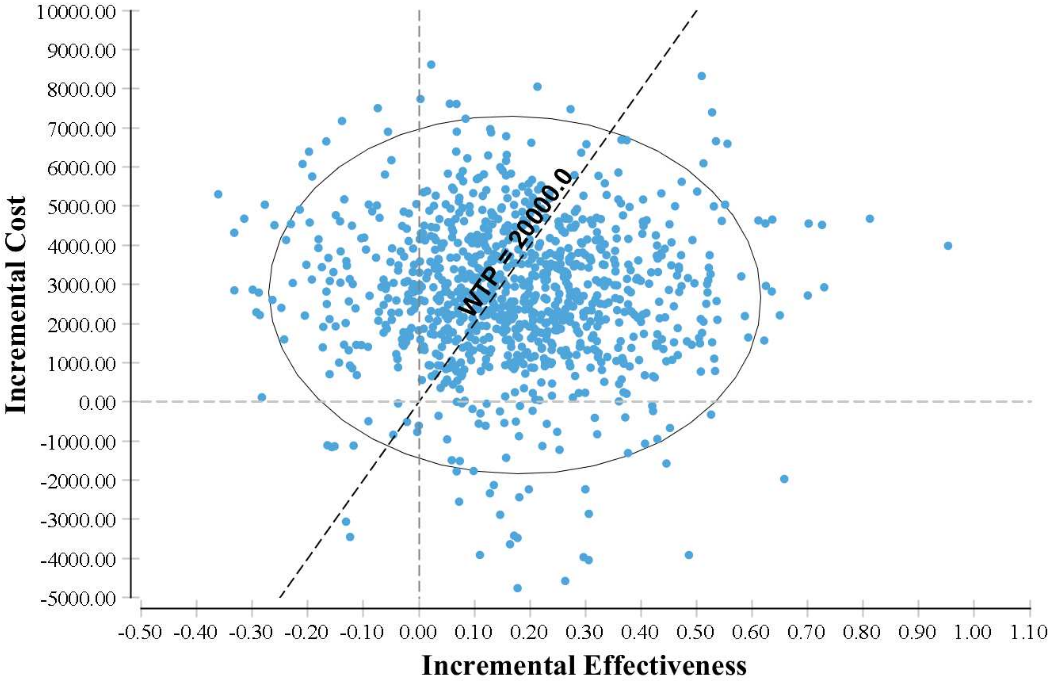

Figure S5: Incremental Cost-Effectiveness Scatterplot; Trabeculectomy vs Medical Treatment (GUI measure) (WTP: Willingness to Pay)

Shabaninejad H, *et al. Br J Ophthalmol* 2024; 108:1210–1215. doi: 10.1136/bjo-2023-323390

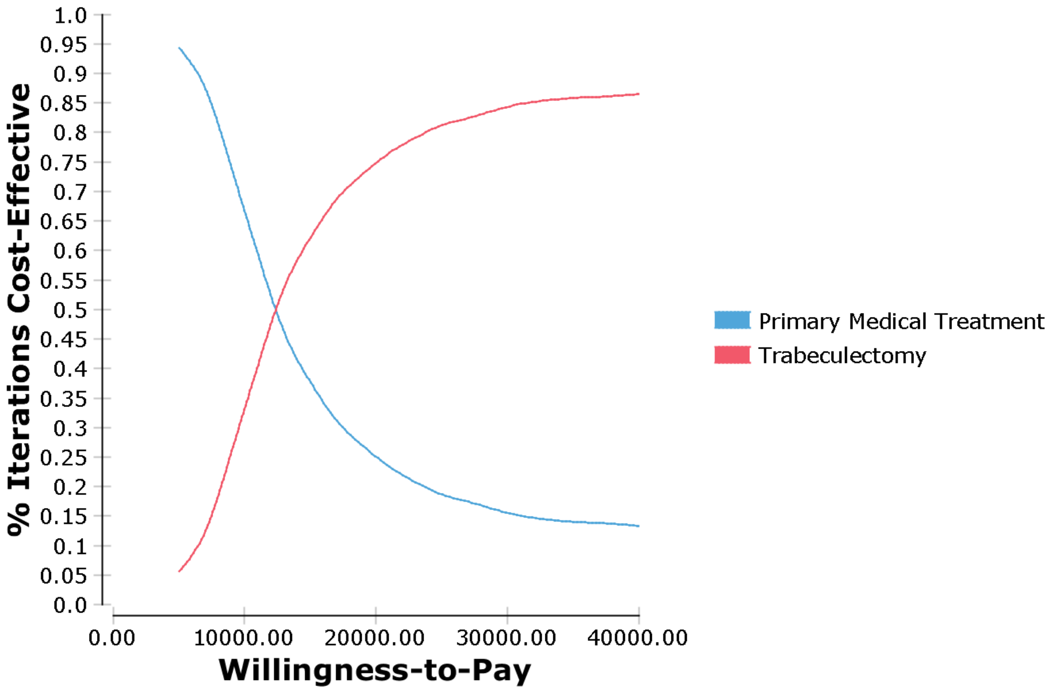

Figure S6: Model-based cost-effectiveness acceptability curve for a life time horizon (30-year); HUI-3 measure

Shabaninejad H, *et al. Br J Ophthalmol* 2024; 108:1210–1215. doi: 10.1136/bjo-2023-323390

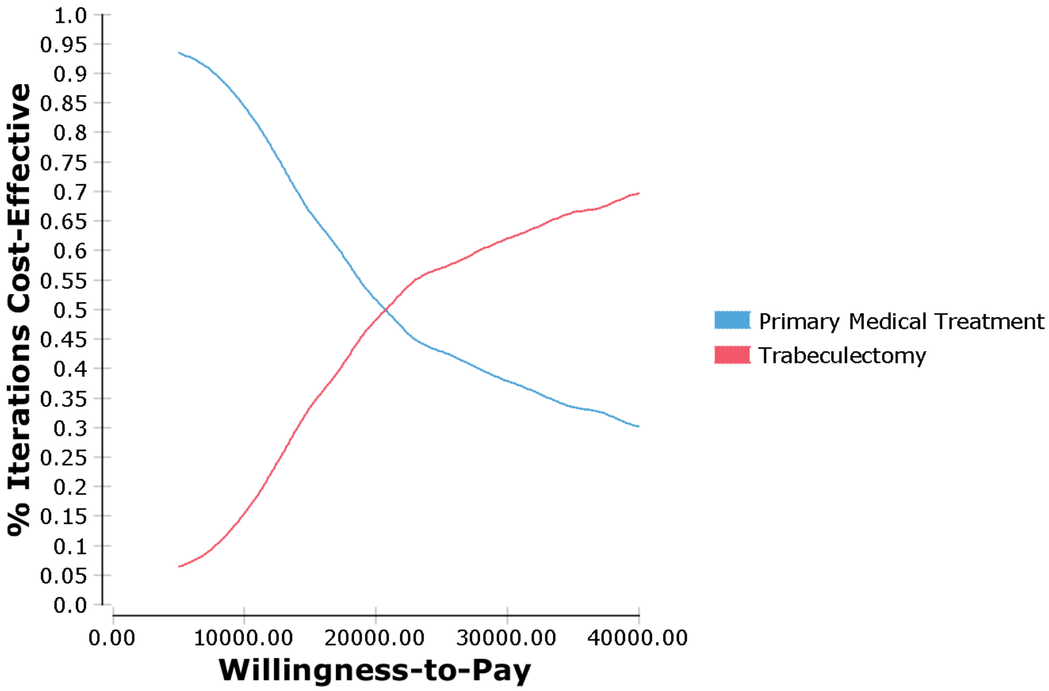

Figure S7: Model-based cost-effectiveness acceptability curve for a life time horizon (30-year); GUI measure
